# Disparities in child mortality trends in two new states of India

**DOI:** 10.1186/1471-2458-13-779

**Published:** 2013-08-27

**Authors:** Mark Minnery, Eliana Jimenez-Soto, Sonja Firth, Kim-Huong Nguyen, Andrew Hodge

**Affiliations:** 1 School of Population Health, Public Health Building, The University of Queensland, Herston Road, Herston, Brisbane, Queensland 4006, Australia

**Keywords:** Health, Child mortality, Health disparities, India, Chhattisgarh, Jharkhand

## Abstract

**Background:**

India has the world’s highest total number of under-five deaths of any nation. While progress towards Millennium Development Goal 4 has been documented at the state level, little information is available for greater disaggregation of child health markers within states. In 2000, new states were created within the country as a partial response to political pressures. State-level information on child health trends in the new states of Chhattisgarh and Jharkhand is scarce. To fill this gap, this article examines under-five and neonatal mortality across various equity markers within these two new states, pre-and post-split.

**Methods:**

Both direct and indirect estimation using pooled data from five available sources were undertaken. Inter-population disparities were evaluated by mortality data stratification of rural–urban location, ethnicity, wealth and districts.

**Results:**

Both states experienced an overall reduction in under-five and neonatal mortality, however, this has stagnated post-2001 and various disparities persist. In cases where disparities have declined, such as between urban–rural populations and low- and high-income groups, this has been driven by modest declines within the disadvantaged groups (i.e. low-income rural households) and stagnation or worsening of outcomes within the advantaged groups. Indeed, rising trends in mortality are most prevalent in urban middle-income households.

**Conclusions:**

The results suggest that rural health improvements may have come at the expense of urban areas, where poor performance may be attributed to factors such as lack of access to quality private health facilities. In addition, the disparities may in part be associated with geographical access, traditional practices and district-level health resource allocation.

## Background

Since the introduction of the Millennium Development Goals (MDGs) in 1990, multiple global and national initiatives have been introduced towards improving maternal and child health [1]. Yet, global estimates of child mortality indicate that less than a quarter of the world is on track to achieve MDG 4 [2] – which calls for a two-thirds reduction in mortality in children younger than 5 years between 1990 and 2015. In 2009, 21 percent of global under-five child deaths and a third of neonatal deaths occurred in India and the country is amongst a group of nations that are unlikely to reach MDG 4 [3-7].

Progress towards the health-related MDGs have tended to focus on improvements in the average health status of the population rather than the distribution of health outcomes [8,9]. While important, assessments of overall child mortality may not completely reflect health conditions in countries with sizeable populations, like India, where the mortality burden is not borne equally within the country. Indeed, a growing body of evidence suggests population averages serve as an inadequate summary measure of a country’s health performance or achievement [10-12]. In India, recent evidence suggests that health policies and efforts to improve coverage of health interventions are not currently reducing inter-population health gaps [13]. Various disparities, related to socioeconomic status, geography and gender, persist [14,15]. For example three quarters of Indian states with an U5MR above the national average are present in the central and the eastern regions [16]. Notwithstanding some studies that have concentrated on rural–urban location and wealth, little has been done to assess recent within-country equity trends, particularly at lower sub-national levels [10-12,17,18].

This article aims to assess the significance of within-country disparities by estimating trends in under-five and neonatal mortality within two states of India, Chhattisgarh and Jharkhand. These states were carved from Madhya Pradesh (Chhattisgarh) and Bihar (Jharkhand) on November 15, 2000 after extended periods of people’s movements and several successful region-wide strikes in response to cultural pressures and political failures. Under the pre-2000 state boundaries, Madyah Pradesh experienced the 21st and 25th highest U5MR out of 25 states in 1992–93 and 1998–99, respectively, while Bihar recorded the 21st highest state-level U5MR in both periods [19,20]. A major rationale for splitting the states was the perceived benefits of smaller sub-national government entities [21]. Yet, barely any evidence exists to assess the success of this experiment via comparisons of the states’ performances pre- and post-separation. In addition, the new states have shown poor indicators in terms of general development and child mortality in contrast to the encouraging progress seen in other parts of India. In 2005–2006 the states had the 25th (Chhattisgarh) and 27th (Jharkhand) highest U5MR out of 29 states in India [16]. These poor performances have been linked to easily avoidable causes of death, such as lack of expanded neonatal and intrapartum care, lack of case management of diarrhoea and pneumonia, and limited addition of new vaccines to immunisation programmes [22]. In 2007–2008 when ranked on the human development index, the states were the 23rd (Chhattisgarh) and 19th (Jharkhand), out of the 23 least developed states of India [23]. Consequently both states remain on the Empowered Action Group (EAG) of Indian states.

In this paper, we estimate disaggregated mortality trends by rural–urban populations, ethnic groups, wealth quintiles and sub-state districts. Little attention has been paid to how the relationships between these factors and health outcomes have changed over time, despite the identification of associations between such characteristics and health disparities [14,24-26]. The creation of the new states adds an additional dimension to these relationships, but to date, has not been examined in the literature. While previous studies have detailed mortality rates for Madhya Pradesh and Bihar pre- and post-2000 and for Chhattisgarh and Jharkhand post-2000 [3], none have included estimates both before and after the creation of Chhattisgarh and Jharkhand. Using district-level data, we are able to provide the first estimates for both states covering the MDG-era of 1990–2015.

As population health disparities can have significant impacts on countries’ developmental progress, the results presented in this study may help to target health resource allocation more effectively. Moreover, the analyses can also provide policy relevant information to key stakeholders on the impact of recent initiatives and guidance towards future programming as the MDG deadline approaches.

## Methods

### Data

We used microdata from a collection of surveys, supplemented with crude death rates from sample registration systems. Table 1 presents an overview of the datasets and their respective samples. The study is based on data available in the public domain.

**Table 1:** Overview of available datasets obtained from surveys in India for Chhattisgarh and Jharkhand, 1990-2008

**Data source**	**Year**	**Data type**		**Sample Size**			**Used for equity marker**					**Comment**
			**CH Women**	**CH CEB**	**JH Women**	**JH CEB**	**ST**	**U/R**	**E**	**W**	**D**	
DLHS-I	1998-1999	SBH	6,456	19,939	14,319	47,672	x	x	x			Converted U5MR to NMR
DLHS-II	2002-2004	CBH	11,163	34388	13,470	45,559	x	x	x	x		
DLHS-III	2007-2008	SBH	18,128	52,710	26,829	78,681	x	x	x		x	Converted U5MR to NMR
DHS-I	1992-1993	CBH										Not used*
DHS-II	1998-1999	CBH										Not used*
DHS-III	2005-2006	CBH	2,638	8,798	2,134	7,280	x	x	x	x		Representative at state level
SRS	1971-2008	Crude death rates					x	x				Data available: CH 2004–2008; JH 2004-2008
Estimation method							S	S	S	D	I	

*Notes*: *DLHS* District level health survey, *DHS* Demographic health survey, *SRS* Sample registration system, *WHS* World Health Survey, *NFHS* National Family Health Survey, *ST* State, *U/R* Urban/rural, *E* Ethnicity, *W* Wealth, *D* District, *S* Summary estimation, *D* Direct estimation, *I* Indirect estimation, *CEB* Children ever born, *U5MR* Under-five mortality rates, *NMR* Neonatal mortality rates, *CH* Chhattisgarh, *JH* Jharkhand.

* Data is only representative at pre-Chhattisgarh and pre-Jharkhand state formations.

The first data source was the Demographic Health Surveys (DHS) series (i.e. the Indian National Family Health Surveys) conducted in 1992–93, 1998–99, and 2005–2006. Similar to other DHS, they provide information on variables related to mortality and fertility, family planning, coverage of maternal and child health services, and socio-economic measures. The sampling design was a systematic, stratified random sample of households, with two stages in rural areas and three stages in urban areas. Details are provided elsewhere [16,19,20].

The second data source used was the District Level Household and Facility Surveys (DLHS) series undertaken in 1998–99, 2002–04, and 2007–08. The DLHS is a collection of district-level representative household surveys, primarily conducted to monitor and assess the implementation and operation of the Reproductive and Child Health program across the districts of India. The DLHS were also undertaken using a systematic, multi-stage stratified sampling design [27-29].

The final source of data used was the Sample Registration System (SRS) dataset. SRS is a sample of birth and death registrations under the Office of the Registrar General of India, and it provides annual estimates of the population, birth rates, fertility, mortality, live births, maternal mortality, life expectancy, death rate, and other indicators at the national and state level and separately for rural and urban place of residence. Generally, the sample design adopted for the SRS is a single-stage stratified random sample [30].

In 2000, the state of Chhattisgarh was formed via the partitioning of 16 south-eastern districts of Madhya Pradesh. At the same time, Jharkhand was carved out of eastern Bihar, including 24 districts, 212 blocks and 32,620 villages. As a result, the 1992–93 and 1998–99 DHS were not usable for these two states since the DHS is only representative at the former-state levels. The DLHS, on the other hand, are representative at the district-level. Consequently, we were able to map the data to fit into the structure of the newly formed states. Similarly, the SRS data were available on a yearly basis, and thus, we were able to account for the changes in the state boundaries.

The pooled datasets resulted in a sample period of 1990–2007. Estimates were produced at the state level and across four equity markers: urban–rural location, ethnicity, wealth, and districts. Our choice of equity markers was informed by the literature and availability of data [9,16,29,31,32]. We should note that SRS only includes measures at the state level and for rural/urban location, while both the DLHS and the DHS waves have data on all the equity markers. As is common with health surveys, data on income and expenditure is not collected. We thus followed previous studies and used principal components analysis to create a wealth index using available information on household assets and housing characteristics [33]. In India the type of assets owned by the rural populations differs considerably from those possessed by the urban populations. Consequently, the asset-based wealth index is derived for both rural and urban areas separately.

### Mortality estimates

The overall methods used to obtain our mortality estimates have been explained in detail elsewhere [34,35]. In short, we follow Rajaratnam and colleagues [36] and derive survey measures of under-five and neonatal mortality from complete birth histories (CBH) and summary birth histories (SBH).

Mortality from CBH was computed by pooling data from all available surveys. Such pooling helps to mitigate some of the known biases associated with CBH [36]. Under-five mortality rates (U5MR) and neonatal mortality rates (NMR) were computed by combining the survival rates from associated age groups and subtracting from one. In the absence of CBH, under-five mortality rates were indirectly estimated from SBH using the combined method developed by Rajaratnam and co-authors [36], which incorporates the cohort-derived and period-derived techniques into a single measure. To convert indirect estimates of U5MR into NMR, the relationships between U5MR and NMR rates were explored using state and sub-state direct estimates of the mortality rates from the other datasets with CBH [37]. The final type of estimated U5MR were derived from the SRS. The SRS only provides under-five crude death rates aggregated across both sexes by state. Accordingly, we applied the commonly used technique outlined by Preston and co-authors to convert the crude death rates to mortality rates [38]. Finally, we computed a summary measure of under-five and neonatal mortality by combining the various estimates previously obtained using Loess regression. The methods used for forecasting U5MR and NMR beyond the sample period are outlined by Murray and colleagues [37].

Three important limitations of our methods should be noted. First, indirect estimates of neonatal rates are converted from estimated U5MR. In the case of wealth, data restrictions imply that computing rates in such a manner would be done with an excessive degree of uncertainty. Consequently, we only calculated direct estimates across wealth groups, which are associated with a lower but still high degree of uncertainty. Second, given the reorganization of administrative districts over time, district-level estimates could only be produced using the most recent DLHS wave. Given the relative size of the sample in this instance, some caution is required when interpreting the results. Thirdly, caution should be taken whenever using nationally representative data to derive birth histories. While anomalies such as improbable birthdates were removed from the final analysis the results presented in this paper remain as estimates, and should be interpreted as such. Other sources have acknowledged the limitations of using such data [39,40]. All statistical analyses described were carried out using two statistical packages, Stata and *R*.

## Results

Health disparities appear across various equity markers within the two states. Additional file 1: Tables S1 and Additional file 1: Table S2 provide U5MR and NMR estimates for both Chhattisgarh and Jharkhand at state level, for rural–urban areas, between three ethnic groupings and by wealth divided into thirds for selected years. Important trends are outlined below.

**Figure 1:**
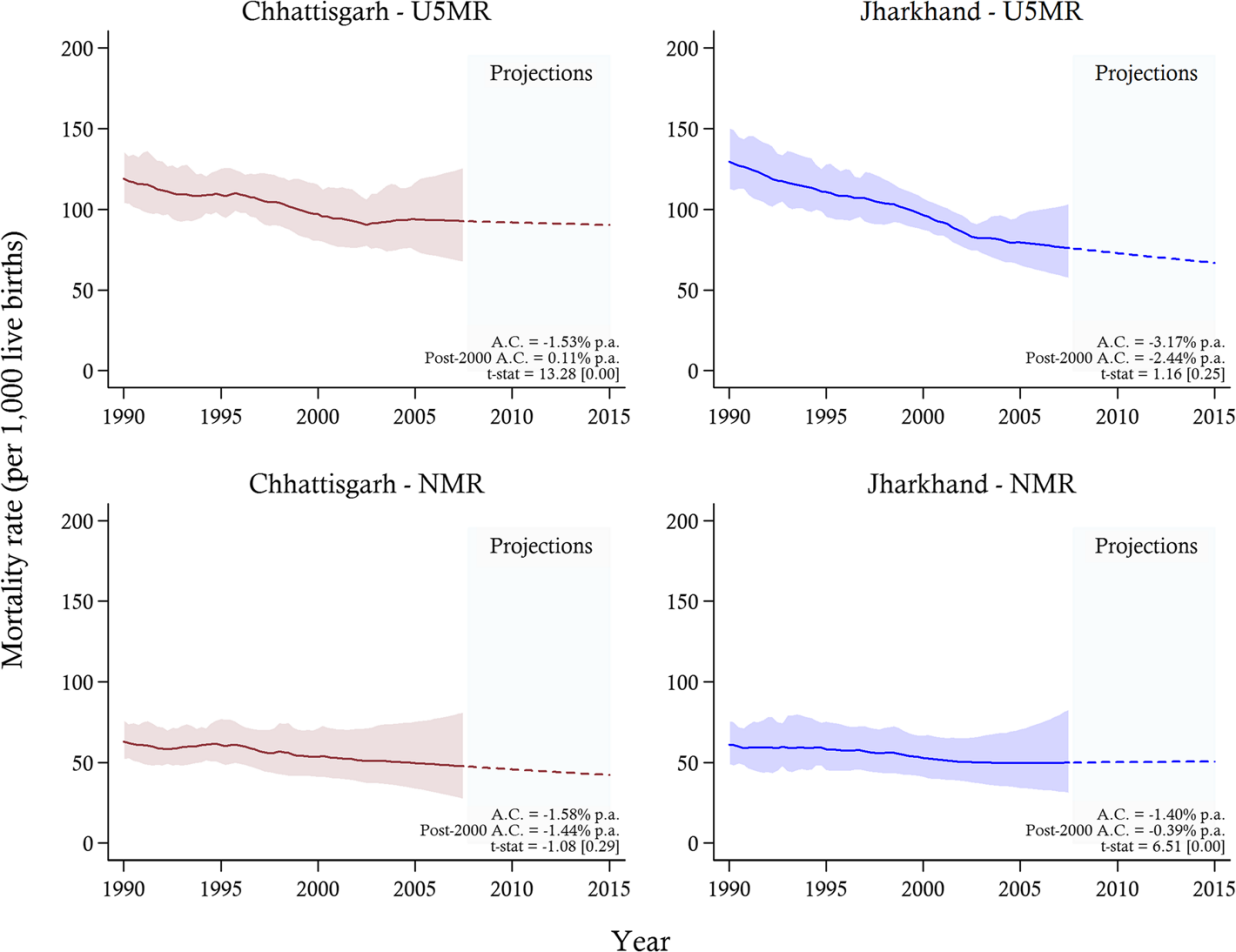
**State-level under-five and neonatal morality rates between 1990 and 2007, with projections towards 2015 and 95% confidence intervals.***Notes*: The solid lines represent the mortality estimates, while the shaded area signifies 95% confidence intervals. Projections are indicated by the dotted-lines. The average annual change (A.C.) in mortality and t-test [*p*-value] for a difference in the A.C. post-2000 are reported. U5MR, under-five mortality rate; NMR, neonatal mortality rate.

U5MRs decreased (see Figure 1) in both states from 119 (95% CI 103–137) and 130 (95% CI 112–150) deaths per 1,000 live births in 1990 to 92 (95% CI 68–124) and 76 (95% CI: 59–102) in 2007 in Chhattisgarh and Jharkhand, respectively. The average annual rates of reduction in U5MRs slowed after the separations of both states in 2001, although the difference is only statistically significant at conventional levels in Chhattisgarh. NMR showed a much smaller reduction of 63 (95% CI: 52–77) to 48 (95% CI: 28–80) deaths per 1,000 live births in Chhattisgarh and 61 (95% CI 49–76) to 50 (95% CI 32–80) in Jharkhand from 1990 to 2007. The stagnating neonatal mortality over the study period contrasts with overall country reductions [6].

**Figure 2:**
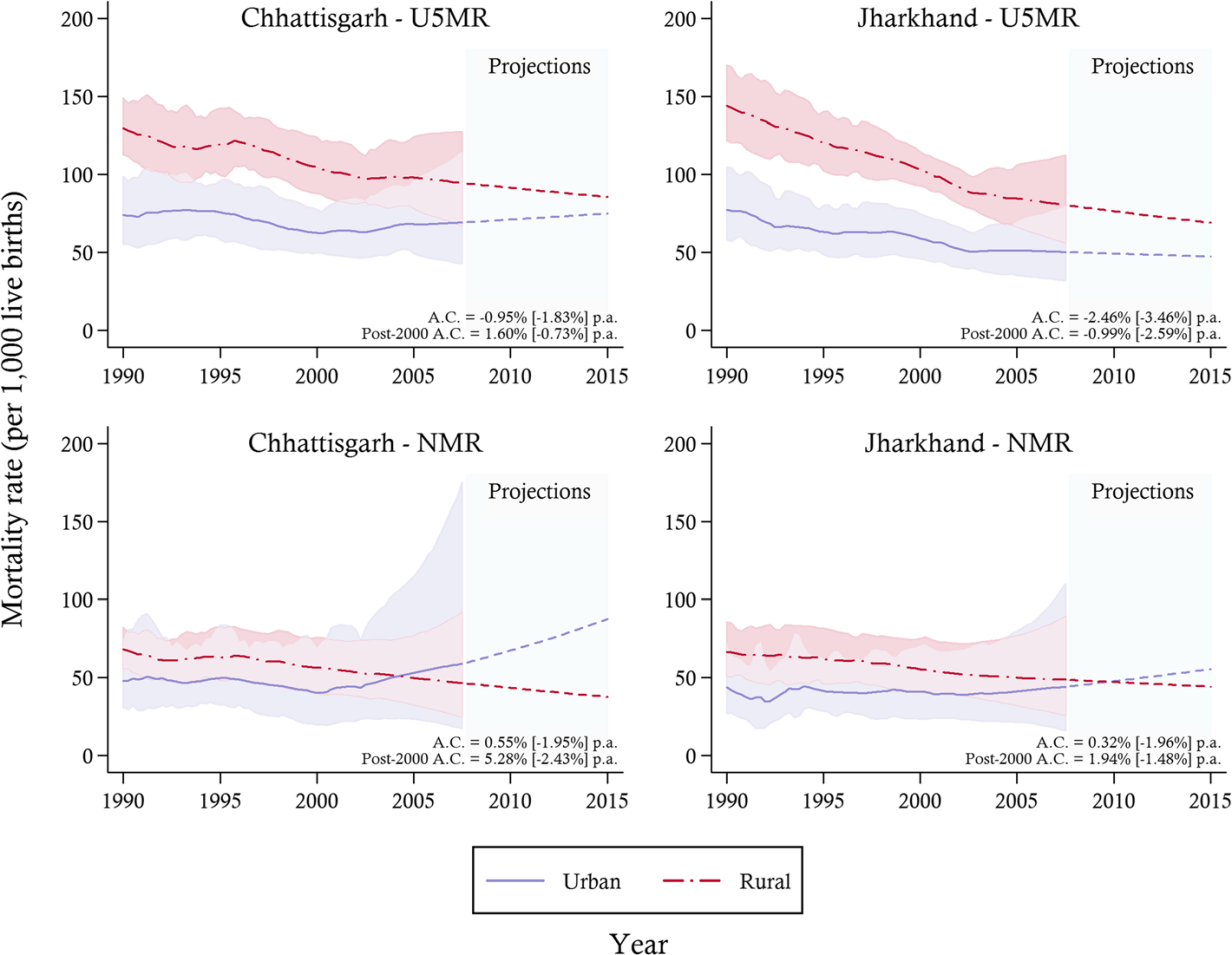
**Under-five and neonatal morality rates stratified by urban–rural location between 1990 and 2007, with projections towards 2015 and 95% confidence intervals.***Notes*: The solid lines represent the mortality estimates, while the shaded area signifies 95% confidence intervals. Projections are indicated by the dotted-lines. The average annual change (A.C.) in mortality is reported for urban [rural] areas. U5MR, under-five mortality rate; NMR, neonatal mortality rate.

Rates of reduction in U5MR were found to favour the rural sector in both states (see Figure 2). Average annual reductions of 3.46% (Jharkhand) and 1.83% (Chhattisgarh) were achieved in rural sectors over the study period compared to 2.46% (Jharkhand) and 0.95% (Chhattisgarh) in the urban areas. Urban areas have experienced increases in NMR in both states, an increase in U5MR in Chhattisgarh and the stagnation in U5MR in Jharkhand towards the end of the study period. Nonetheless mortality rates remained highest in the rural sector with rural / urban rates of under-five mortality estimated at 95 (95% CI: 69–127) / 69 (95% CI: 44–110) in Chhattisgarh and 81 (95% CI: 58–111) / 50 (95% CI: 33–77) Jharkhand in 2007. The estimates suggest that convergence of rural and urban mortality will largely be due not to improvements in child health in rural areas but to stagnation and poor performance in urban areas.

**Figure 3:**
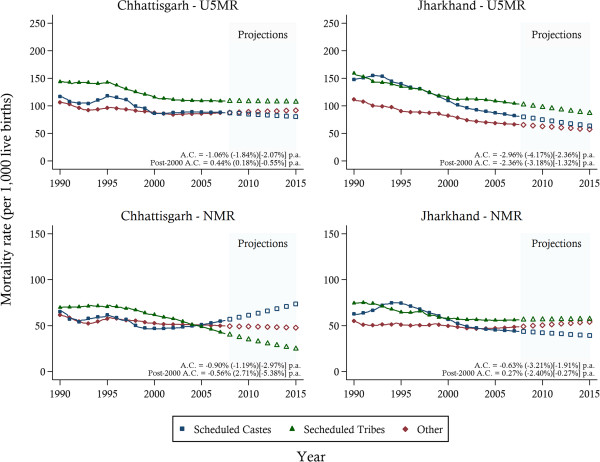
**Under-five mortality trends between 1990 and 2005 and projections towards 2015 by ethnic groups.***Notes*: The solid lines represent the mortality estimates. Projections are indicated by the hollow symbols. The average annual change (A.C.) in mortality is reported for Other (Scheduled Caste) [Scheduled Tribes] ethnic groups. U5MR, under-five mortality rate; NMR, neonatal mortality rate.

Disparities were also prevalent amongst ethnic groups (Figure 3). Mortality rates were the highest in both Chhattisgarh and Jharkhand amongst the Scheduled Tribes (STs). This sub-population experienced the highest rates of under-five mortality in both states and in Jharkhand they also experienced the slowest rate of reduction amongst the three ethnic groups. NMRs for STs showed marked reductions post-1998 in Chhattisgarh and pre-2000 in Jharkhand. Of additional note is the trend of NMR increase in Chhattisgarh post-2000 amongst the Scheduled Caste (SCs) group which may reflect changing patterns of U5MR distribution within the state.

**Figure 4:**
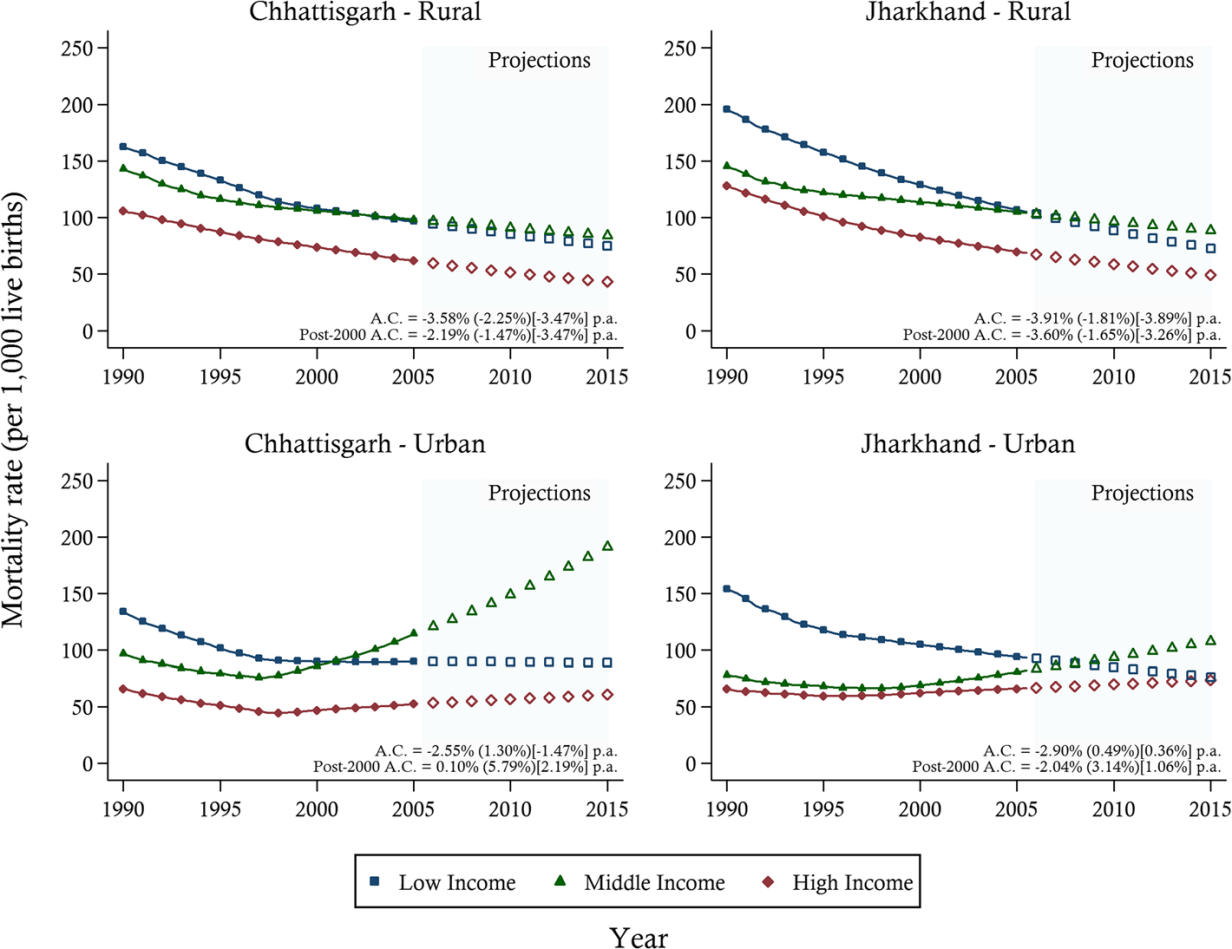
**Under-five mortality trends between 1990 and 2005 and projections towards 2015 by wealth groups, stratified by urban–rural location.***Notes*: The solid lines represent the mortality estimates. Projections are indicated by the hollow symbols. The average annual change (A.C.) in mortality is reported for Low (Middle) [High] income groups.

**Figure 5:**
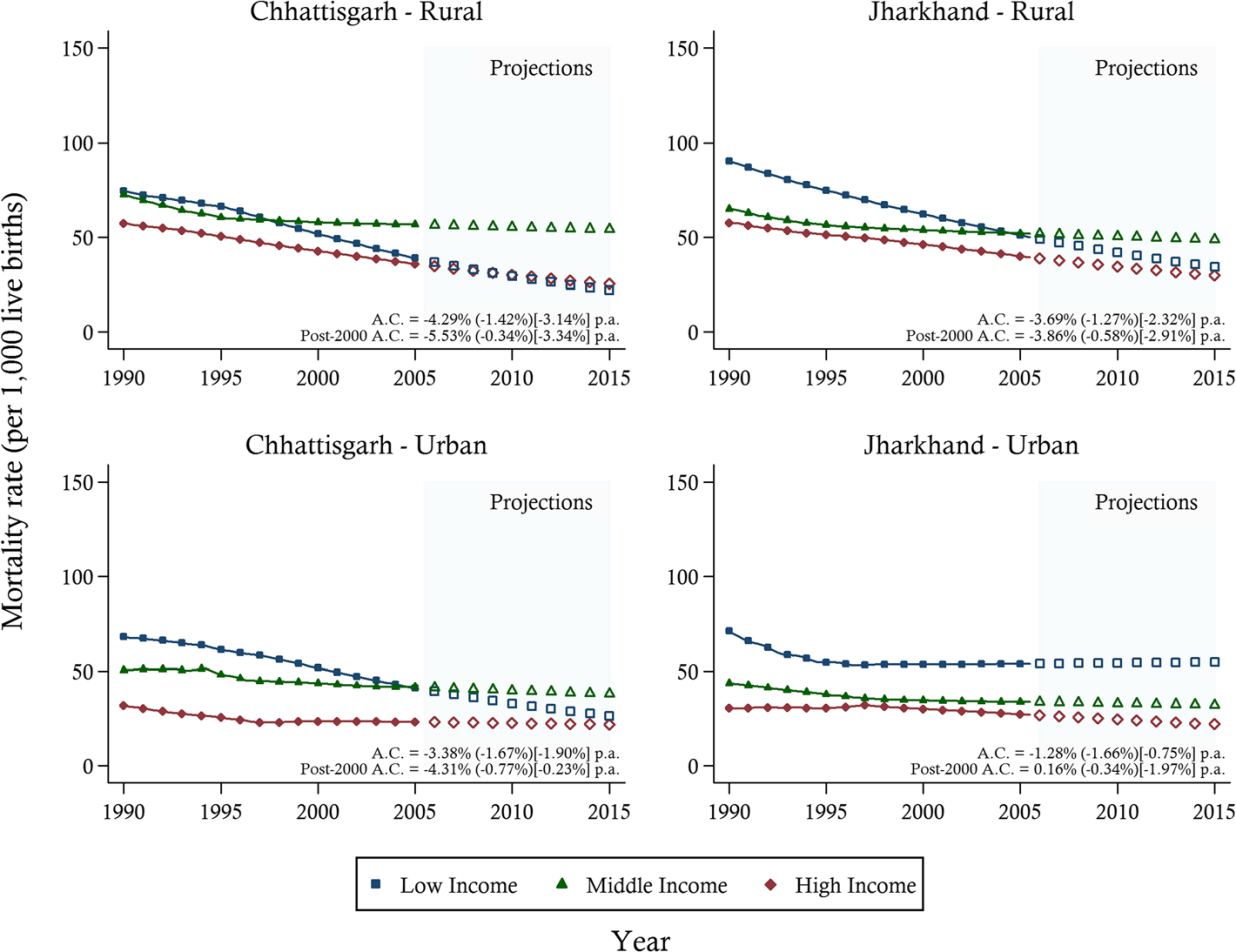
**Neonatal mortality trends between 1990 and 2005 and projections towards 2015 by wealth groups, stratified by urban–rural location.***Notes*: The solid lines represent the mortality estimates. Projections are indicated by the hollow symbols. The average annual change (A.C.) in mortality is reported for Low (Middle) [High] income groups.

Under-five and neonatal mortality estimates for wealth groups are presented in Figures 4 (for U5MR) and Figure 5 (for NMR), stratified by urban–rural location. While some convergence between the lowest- and highest-income groups was evident in both states, the middle-income group showed limited reductions in mortality rates, particularly in urban areas. A sharp increase in urban U5MRs was found towards the middle and end of the study period for the middle-income group. This pattern was echoed in NMR in Chhattisgarh. In Jharkhand, however, NMRs, after an initial early decrease particularly in the lowest income group, experienced stagnation across all wealth groups.

**Figure 6:**
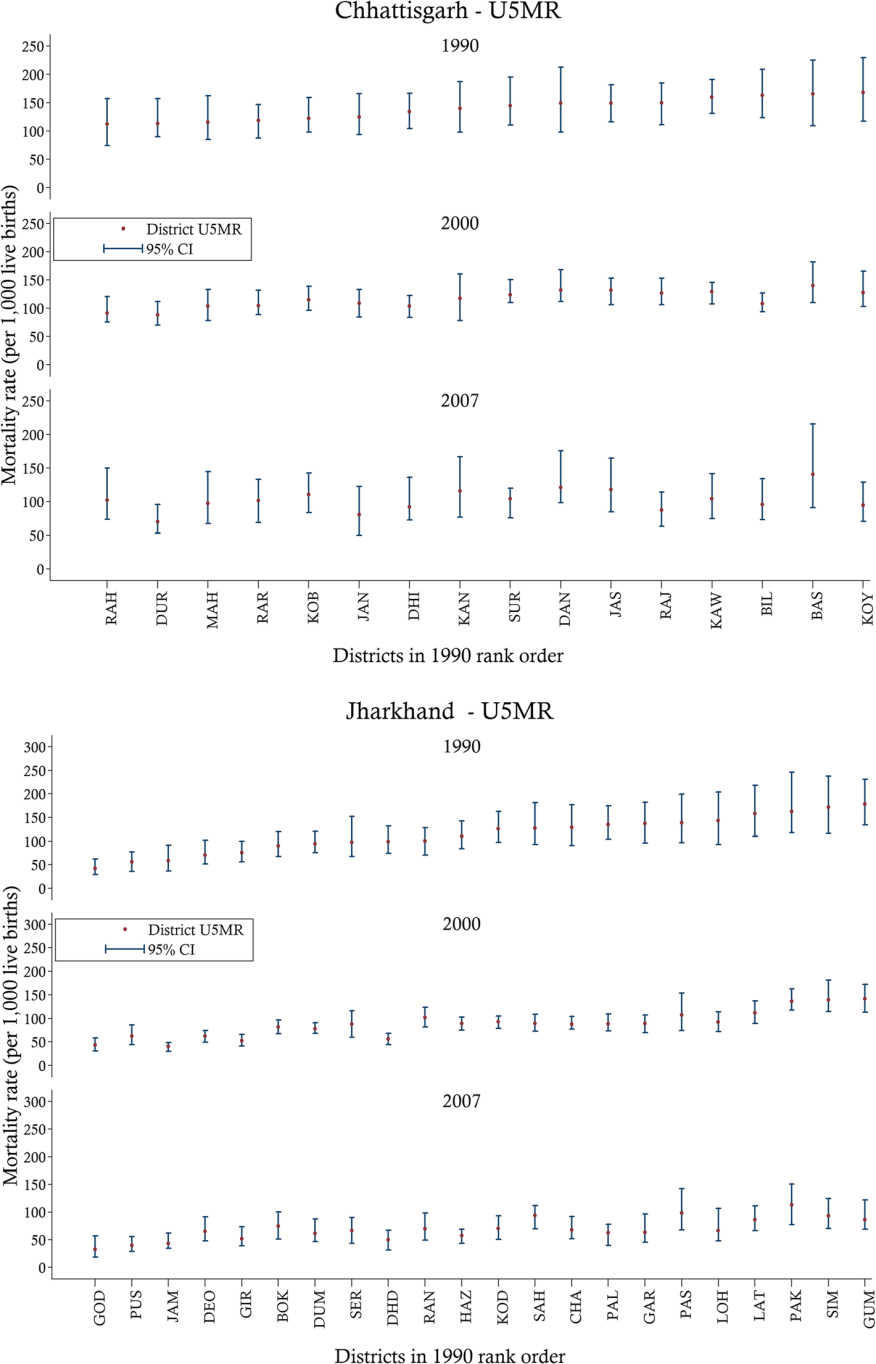
**District-level under-five mortality rates with 95% confidence intervals for selected years.***Notes*: District codes and names are as follows: Chhattisgarh – BAS, Bastar; BIL, Bilaspur; DAN, Dantewada DHI, Dhamtari; DUR, Durg; JAN, Janjgir-Champa; JAS, Jashpur; KAN, Kanker; KAW, Kawardha; KOB, Korba; KOY, Koriya; MAH, Mahasamund; RAH, Raigarh; RAR, Raipur; RAJ, Rajnandgaon; SUR, Surguja. Jharkhard – BOK, Bokaro; CHA, Chatra; DEO, Deoghar; DHD, Dhanbad; DUM, Dumka; GAR, Garhwa; GIR, Giridih; GOD, Godda; GUM, Gumla; HAZ, Hazaribagh; JAM, Jamtara; KOD, Kodarma; LAT, Latehar; LOH, Lohardaga; PAK, Pakaur; PAL, Palamu; PAS, Pashchimi Singhbhum; PUS, Purbi Singhbhum; RAN, Ranchi; SAH, Sahibganj; SER, Seraikela (Saraikela-Kharsawan); SIM, Simdega. U5MR, under-five mortality; CI, confidence interval.

District-level estimates are presented in Figure 6 and reveal the geo-spatial distribution of U5MRs within the states. In both states the pattern of change was non-uniform over time and significant variations in U5MR were experienced across the districts. While some districts were able to make reductions in U5MRs, others stagnated, and no clear association between the rate of change and the initial level of under-five mortality was evident. Estimates also showed a large disparity within states, with the worst performing districts experiencing U5MR in 1990 of 169 (95% CI 118 230) and 178 (95% CI 134–231) while the best districts achieved rates of 112 (95% CI 74–157) and 42 (95% CI 29–62) deaths per 1,000 live births in Chhattisgarh and Jharkhand, respectively.

## Discussion

National levels of child deaths throughout India are declining steadily [4] but state-level results disaggregated by key equity markers demonstrate that the distribution of these reductions are unequal. Chhattisgarh and Jharkhand pre- and post-separation show markedly lower rates of reduction than the national average for both U5MRs and NMRs and within-state disparities are considerable. Across urban/rural divides, ethnic groups, wealth groups and districts, child health improvements vary at levels indicative of differential levels of care.

A pattern of stagnation in U5MRs for Chhattisgarh and Jharkhand after 2001 may in part be explained by the formation of new states. As health resources are primarily administered at the state level in India [13] the disruption and fragmentation of the management of services through the splitting of the states into their new administrative areas may have been a contributing factor. Direct investigation of this hypothesis, however, is beyond the scope of this paper and would require further analysis in order to draw stronger conclusions.

Rural sectors in both states have made significant reductions in both U5MRs and NMRs. This may, however, have come at the cost of urban health. It has been suggested that urban children face distinct difficulties in access to health services while also being vulnerable to other risk factors such as the effects of crowding and indoor air pollution [41]. These factors may partly explain the disappointing progress in urban child health with estimated increase in urban U5MRs and NMRs in both states towards the end of our study period.

An influx of the rural sector population into urban centres creating an increase in the urban poor and middle-income population may also help to explain worsening urban health markers [42]. Figures from census data [30] indicate more than 6 percent of the Bihar urban population (at the time encompassing Jharkhand) between 1991 and 2001 had migrated from rural areas. With livelihood opportunities major drivers of migration [42], poorer and low-health status rural households seem to be more likely to migrate to urban areas than those that are better off. As such, the lower health status of the migrating population may be creating a literal shift of population health from rural to urban areas. Unfortunately the cross-sectional nature of the available data does not allow us to trace regional movements of particular families, and thus, we cannot formally test issues related to migration. Programs initiated under the National Rural Health mission, such as the implementation of Accredited Social Health Activists (ASHA), may also offer an explanation of the differing progress seen between rural and urban areas after 2005. The central role of the ASHA in care seeking and in some instances administering care particularly in low income rural communities may explain in part the greater performance of this sector post 2005 and is an important consideration for future research [43-45]. However the effects of individual programs under the NRHM on U5MR and NMR cannot yet be determined given that the estimates produced in this study only cover a short period under which these programs have been implemented (2005 – 2007).

The increasing trend in child health amongst urban middle-income households at first glance appears to be counterintuitive but is potentially linked to the current private-public health system debate [46]. The privatisation of health services in urban centres [47] poses financial barriers, which prevents access to quality MNCH services. It has been shown that the patient perceived quality of care in Indian private institutions is high while the public care is perceived as slow and inefficient [48]. Consequently, the middle-income group, who have the means to access private care, may favour the private sector [48]. However, it has also been shown that patient outcomes and quality of care in the private sector is lower than that of the public sector in India and other lower-middle income countries [46,49]. This suggests that middle-income households may be mistakenly accessing private institutions of low quality resulting in poor patient outcomes [50]. Waitzkin and colleagues [51] also show that the likelihood of worse care and outcomes is higher amongst poorer people seeking access at private institutions. This too suggests that those that are not wealthy but still have the means to access private care may be receiving sub-standard treatment. Studies within other states of India have indicated an overall preference for unrecognised medical practitioners amongst key groups such as urban slum/remote rural residents [52-54] which may have deleterious effects on health outcomes [55]. Nevertheless, the use of unrecognised medical practitioners has not been widely researched in relation to accessing from different wealth groups in India but remains an important consideration in the private-public debate more generally and an area in need of further research.’

Both Chhattisgarh and Jharkhand have seen unequal reductions amongst ethnic groups, with the ST generally lagging behind the other groups. As noted by the directorate of Census Operations 2001 [30] STs and SCs in Chhattisgarh overwhelmingly reside rurally in villages. Remote villages often lack access to health services resulting in low coverage of interventions targeted at improving child health [56,57]. Traditional beliefs in herbal treatment may also create barriers to utilisation of formal health services [58-60]. Using the NFHS-III, Pradhan and Arokiasamy [32] showed that belonging to a scheduled tribe or a scheduled caste, residing in a rural location, and mother's education level all contributed heavily to child (and maternal) mortality. STs and SCs also reside primarily in rural locations in Jharkhand, with high concentrations living in villages, and thus, may face similar barriers to health services to Chhattisgarh [56]. On a positive note, the highly concentrated areas of ST populations may present an opportunity for targeting increased intervention coverage for well-designed programmes similar to the Navajyoti scheme implemented in Orissa from 2005 [61].

District level analysis shows stark differences throughout both states. Geography-related disparities may be in part explained by the distribution and relative resources available for each district [62], although little evidence is available on this. In developing settings, geographical disparities are underscored by systemic poverty and disadvantage [63,64]. This compounds problems associated with weak local governmental capacity to provide quality health services [65]. In addition, both Chhattisgarh and Jharkhand have experienced decades of civil unrest and undoubtedly such socio-political issues will have had strong, differential impacts on mortality outcomes of different districts and ethnic groups.

The main purpose of this study was to systematically collate evidence on levels and trends of child mortality for different sub-populations in Chhattisgarh and Jharkhand over the MDG time period. Yet, several important limitations remain. Firstly, under-reporting of deaths by mothers and recall bias may have had substantial effects on directly estimated child mortality rates. To mitigate this problem we have pooled data from multiple surveys and declined to create estimates for periods with 5,000 or fewer person-months of observations. Secondly, the limitations of indirect estimation are well-known and have been documented in studies such as that by Rajaratnam and co-authors [36]. Of particular concern is the necessity for inferring statistical information, such as birth and death location and time, from observed patterns in CBH surveys. This can lead to a reliance on generalised patterns across time and states. Local regression methods are used to minimise the impact of these generalisations. Thirdly, limited available observation numbers, particularly towards the end of the study period, tends to lead to large sampling errors, which implies that caution is required when interpreting results. Lastly, any recent impacts achieved from efforts aimed at targeting specific sub-populations or reducing child mortality in certain areas are not directly captured in our forecasts.

## Conclusion

Progress has been made in the new states of Chhattisgarh and Jharkhand in reducing child and neonatal mortality, but disparities across different socioeconomic and geographical markers remain. Investments that focus on increasing quality health services to identify disadvantaged populations are necessary to reduce disparities and produce better child mortality figures for the two states. Research on how best to extend health services to these disadvantaged populations is warranted. In addition, investments in the quality of care offered by the both the private and public health systems throughout the nation may be required in order to ensure continued progress in child health is achieved equally within India in the post-MDG era.

## Competing interests

The authors declare that they have no competing interests.

## Authors’ contributions

MM provided interpretation and prepared the manuscript. EJS and SF initiated the study. EJS and KHN participated in the research design and preformed data analysis and interpretation. AH was involved in the data analysis and interpretation of the data. AH and SF contributed to the critical revision of the manuscript. All authors read and approved the final manuscript.

## Pre-publication history

The pre-publication history for this paper can be accessed here:

http://www.biomedcentral.com/1471-2458/13/779/prepub

## Supplementary Material

Additional file 1: Table S1 Estimated under-five mortality rates (per 1,000 live births), with 95% confidence intervals, for selected years. **Table S2.** Estimated neonatal mortality rates (per 1,000 live births), with 95% confidence intervals, for selected years. Click here for file
