# Supplementary material for: Disparities in child mortality trends in two new states of India
Source: BMC Public Health. 2013 Aug 27;13:779. doi: 10.1186/1471-2458-13-779 (PMC3765884; doi:10.1186/1471-2458-13-779)
Supplement: Additional file 1: Table S1 — Estimated under-five mortality rates (per 1,000 live births), with 95% confidence intervals, for selected years. Table S2. Estimated neonatal mortality rates (per 1,000 live births), with 95% confidence intervals, for selected years. [file 1471-2458-13-779-S1.docx]

**Additional file**

**Additional file1:** Table S1: Estimated under-five mortality rates (per 1,000 live births), with 95% confidence intervals, for selected years

| **Equity marker** | **1990** | | | **1995** | | | **2000** | | | **2005/2007^*^** | | |
| --- | --- | --- | --- | --- | --- | --- | --- | --- | --- | --- | --- | --- |
|  | U5MR | 95% C.I. | | U5MR | 95% C.I. | | U5MR | 95% C.I. | | U5MR | 95% C.I. | |
| **CH** | 119.08 | (103.46; | 136.79) | 108.98 | (95.42; | 125.79) | 96.88 | (80.04; | 115.75) | 92.86 | (68.30; | 124.15) |
| **Urban/Rural** |  |  |  |  |  |  |  |  |  |  |  |  |
| Rural | 129.62 | (112.51; | 148.8) | 119.07 | (99.54; | 142.32) | 104.91 | (87.85; | 124.10) | 95.34 | (69.50; | 127.11) |
| Urban | 74.04 | (55.14; | 98.65) | 75.61 | (58.16; | 96.15) | 62.64 | (49.51; | 77.46) | 68.82 | (43.81; | 110.16) |
| **Ethnicity** |  |  |  |  |  |  |  |  |  |  |  |  |
| Scheduled Caste | 116.79 | (91.02; | 146.40) | 118.04 | (98.93; | 147.56) | 86.17 | (66.72; | 109.65) | 87.93 | (59.18; | 137.13) |
| Scheduled Tribe | 143.86 | (119.05; | 173.81) | 142.61 | (116.26; | 171.35) | 115.88 | (89.98; | 143.09) | 108.90 | (74.81; | 153.28) |
| Other | 106.46 | (91.73; | 124.35) | 96.32 | (80.87; | 116.74) | 88.13 | (73.17; | 106.26) | 87.23 | (58.80; | 119.45) |
| **Wealth** |  |  |  |  |  |  |  |  |  |  |  |  |
| *Rural* |  |  |  |  |  |  |  |  |  |  |  |  |
| Low Income. | 162.75 | (119.48; | 218.32) | 132.97 | (98.10; | 175.58) | 108.10 | (68.54; | 163.39) | 96.60 | (54.08; | 163.91)* |
| Middle Income. | 143.20 | (93.96; | 210.42) | 116.56 | (80.43; | 166.45) | 106.20 | (73.85; | 149.78) | 98.48 | (63.89; | 145.79)* |
| High Income. | 106.10 | (68.41; | 159.65) | 87.44 | (56.90; | 132.08) | 73.87 | (47.98; | 110.55) | 61.78 | (35.88; | 100.81)* |
| *Urban* |  |  |  |  |  |  |  |  |  |  |  |  |
| Low Income | 134.03 | (85.01; | 204.86) | 101.50 | (65.44; | 153.48) | 89.89 | (49.06; | 155.74) | 90.03 | (40.68; | 186.38)* |
| Middle Income | 96.92 | (41.03; | 220.06) | 79.51 | (34.90; | 178.58) | 86.21 | (30.52; | 225.30) | 114.53 | (32.89; | 348.90)* |
| High Income | 65.86 | (29.51; | 131.68) | 51.40 | (20.57; | 120.81) | 47.12 | (12.13; | 170.07) | 52.93 | (10.12; | 280.96)* |
|  |  |  |  |  |  |  |  |  |  |  |  |  |
| **JH** | 129.57 | (112.46; | 150.73) | 110.62 | (94.93; | 128.33) | 96.55 | (86.88; | 107.26) | 76.68 | (58.49; | 102.07) |
| **Urban/Rural** |  |  |  |  |  |  |  |  |  |  |  |  |
| Rural | 143.83 | (121.82; | 169.93) | 120.56 | (102.37; | 140.39) | 103.21 | (92.71; | 114.66) | 81.26 | (58.16; | 110.99) |
| Urban | 77.52 | (58.17; | 104.76) | 63.10 | (48.51; | 79.93) | 59.01 | (45.84; | 74.54) | 50.35 | (32.67; | 77.39) |
| **Ethnicity** |  |  |  |  |  |  |  |  |  |  |  |  |
| Scheduled Caste | 147.83 | (118.44; | 184.55) | 140.10 | (114.45; | 167.12) | 109.44 | (89.01; | 134.94) | 82.31 | (55.44; | 119.33) |
| Scheduled Tribe | 159.18 | (129.33; | 193.57) | 134.97 | (114.34; | 158.95) | 114.98 | (93.26; | 140.97) | 104.48 | (72.41; | 149.28) |
| Other | 112.00 | (97.33; | 132.56) | 90.47 | (78.45; | 101.41) | 82.23 | (71.32; | 95.53) | 66.64 | (50.44; | 91.62) |
| **Wealth** |  |  |  |  |  |  |  |  |  |  |  |  |
| *Rural* |  |  |  |  |  |  |  |  |  |  |  |  |
| Low Income | 195.58 | (151.66; | 250.42) | 157.82 | (124.99; | 201.39) | 128.88 | (94.15; | 174.09) | 106.89 | (70.91; | 155.72)* |
| Middle Income | 145.51 | (106.83; | 192.55) | 122.13 | (98.87; | 148.67) | 113.95 | (93.40; | 138.14) | 104.97 | (80.82; | 134.64)* |
| High Income | 128.51 | (87.40; | 184.43) | 101.12 | (70.45; | 141.99) | 82.87 | (56.55; | 121.45) | 69.95 | (43.30; | 114.92)* |
| *Urban* |  |  |  |  |  |  |  |  |  |  |  |  |
| Low Income | 154.02 | (94.60; | 243.54) | 117.72 | (72.64; | 181.05) | 104.84 | (66.10; | 161.58) | 94.24 | (55.45; | 164.38)* |
| Middle Income | 78.39 | (40.75; | 143.99) | 68.23 | (34.32; | 131.95) | 69.20 | (34.48; | 136.24) | 80.90 | (33.60; | 191.41)* |
| High Income | 66.06 | (31.34; | 139.52) | 59.86 | (26.20; | 139.81) | 62.44 | (22.90; | 161.10) | 66.28 | (18.38; | 205.45)* |

*Notes*: * The estimates from the more recent year are represented in the final column. For wealth quintiles the most recent year is 2005, for all other equity markers the year is 2007.

**Additional file 1**:Table S2: Estimated neonatal mortality rates (per 1,000 live births), with 95% confidence intervals, for selected years

| **Equity marker** | **1990** | | | **1995** | | | **2000** | | | **2005/2007^*^** | | |
| --- | --- | --- | --- | --- | --- | --- | --- | --- | --- | --- | --- | --- |
|  | NMR | 95% C.I. | | NMR | 95% C.I. | | NMR | 95% C.I. | | NMR | 95% C.I. | |
| **CH** | 62.95 | (51.62; | 76.93) | 60.93 | (48.91; | 77.56) | 53.55 | (40.64; | 71.52) | 48.03 | (28.43; | 79.97) |
| **Urban/Rural** |  |  |  |  |  |  |  |  |  |  |  |  |
| Rural | 68.30 | (55.99; | 82.39) | 62.94 | (48.42; | 82.25) | 56.45 | (40.01; | 75.58) | 47.19 | (26.53; | 88.06) |
| Urban | 47.69 | (30.75; | 74.86) | 49.69 | (32.55; | 83.50) | 40.10 | (19.59; | 68.99) | 57.91 | (18.79; | 159.28) |
| **Ethnicity** |  |  |  |  |  |  |  |  |  |  |  |  |
| Scheduled Caste | 65.14 | (36.48; | 118.45) | 61.65 | (38.68; | 94.22) | 46.98 | (24.61; | 83.18) | 54.74 | (17.50; | 169.28) |
| Scheduled Tribe | 69.77 | (46.60; | 103.66) | 70.81 | (47.24; | 103.77) | 61.93 | (35.62; | 104.82) | 42.98 | (12.88; | 107.89) |
| Other | 61.67 | (47.38; | 79.98) | 57.55 | (43.11; | 79.69) | 52.81 | (36.36; | 75.61) | 49.93 | (25.87; | 98.78) |
| **Wealth** |  |  |  |  |  |  |  |  |  |  |  |  |
| *Rural* |  |  |  |  |  |  |  |  |  |  |  |  |
| Low Income | 74.29 | (44.78; | 117.14) | 66.22 | (41.79; | 101.99) | 51.84 | (29.91; | 87.81) | 38.84 | (20.10; | 73.45)* |
| Middle Income | 72.48 | (46.86; | 109.91) | 60.50 | (37.82; | 94.97) | 57.95 | (36.29; | 92.28) | 56.78 | (34.14; | 97.29)* |
| High Income | 57.29 | (31.99; | 99.13) | 50.52 | (30.26; | 85.87) | 42.74 | (24.83; | 75.56) | 35.91 | (18.78; | 69.53)* |
| *Urban* |  |  |  |  |  |  |  |  |  |  |  |  |
| Low Income | 68.10 | (31.90; | 138.10) | 61.43 | (28.73; | 128.14) | 51.69 | (21.71; | 118.32) | 41.18 | (14.82; | 107.20)* |
| Middle Income | 50.56 | (12.03; | 183.80) | 48.26 | (10.65; | 207.23) | 43.68 | (8.01; | 210.62) | 41.77 | (5.77; | 245.79)* |
| High Income | 31.96 | (11.33; | 93.68) | 25.63 | (7.09; | 89.84) | 23.57 | (4.31; | 116.57) | 23.39 | (3.12; | 156.49)* |
|  |  |  |  |  |  |  |  |  |  |  |  |  |
| **JH** | 61.22 | (48.71; | 76.20) | 58.13 | (44.77; | 75.77) | 52.87 | (40.61; | 67.08) | 49.76 | (31.52; | 80.04) |
| **Urban/Rural** |  |  |  |  |  |  |  |  |  |  |  |  |
| Rural | 66.66 | (51.02; | 85.64) | 61.58 | (46.14; | 81.59) | 55.37 | (40.75; | 75.03) | 48.75 | (27.19; | 86.00) |
| Urban | 43.75 | (27.07; | 72.07) | 41.31 | (28.68; | 61.84) | 40.95 | (23.31; | 70.92) | 43.59 | (17.68; | 101.26) |
| **Ethnicity** |  |  |  |  |  |  |  |  |  |  |  |  |
| Scheduled Caste | 62.77 | (37.86; | 97.43) | 74.44 | (51.02; | 112.52) | 56.79 | (36.14; | 94.63) | 44.11 | (16.54; | 108.20) |
| Scheduled Tribe | 74.55 | (46.81; | 115.99) | 64.80 | (44.82; | 91.76) | 57.84 | (36.48; | 91.28) | 56.17 | (19.80; | 128.65) |
| Other | 55.21 | (40.55; | 76.19) | 50.97 | (35.82; | 71.76) | 49.80 | (37.30; | 63.73) | 48.52 | (28.13; | 84.16) |
| **Wealth** |  |  |  |  |  |  |  |  |  |  |  |  |
| *Rural* |  |  |  |  |  |  |  |  |  |  |  |  |
| Low Income | 90.23 | (61.61; | 128.70) | 74.79 | (52.88; | 102.39) | 62.65 | (41.96; | 89.32) | 51.03 | (31.49; | 80.00) |
| Middle Income | 65.08 | (41.50; | 99.07) | 56.50 | (41.53; | 76.62) | 53.78 | (39.00; | 73.96) | 52.09 | (36.07; | 75.86) |
| High Income | 57.63 | (33.63; | 95.45) | 51.33 | (32.10; | 81.48) | 46.24 | (28.74; | 75.29) | 39.98 | (22.29; | 71.61) |
| *Urban* |  |  |  |  |  |  |  |  |  |  |  |  |
| Low Income | 71.09 | (34.68; | 135.91) | 54.56 | (25.64; | 107.90) | 53.42 | (24.89; | 106.61) | 53.79 | (20.84; | 124.24) |
| Middle Income | 43.82 | (15.55; | 122.44) | 37.88 | (11.91; | 121.99) | 34.70 | (10.02; | 114.52) | 33.96 | (7.68; | 133.05) |
| High Income | 30.54 | (8.53; | 101.74) | 30.63 | (8.03; | 111.15) | 30.13 | (8.66; | 100.00) | 27.32 | (7.67; | 89.20) |

*Notes*: * The estimates from the more recent year are represented in the final column. For wealth quintiles the most recent year is 2005, for all other equity markers the year is 2007.
